# Protein-Protein Interaction Network Analysis Revealed a New Prospective of Posttraumatic Stress Disorder

**DOI:** 10.22086/gmj.v0i0.1137

**Published:** 2018-05-29

**Authors:** Farshad Okhovatian, Mostafa Rezaei Tavirani, Mohammad Rostami-Nejad, Sina Rezaei Tavirani

**Affiliations:** ^1^Physiotherapy Research Center, Shahid Beheshti University of Medical Sciences, Tehran, Iran; ^2^Proteomics Research Center, Faculty of Paramedical Sciences, Shahid Beheshti University of Medical Sciences, Tehran, Iran; ^3^Gastroenterology and Liver Diseases Research Center, Research Institute for Gastroenterology and Liver Diseases, Shahid Beheshti University of Medical Sciences, Tehran, Iran

**Keywords:** Posttraumatic Stress Disorder, Protein-Protein Interaction Network, Cytoscape, ClueGO, Biomarker Panel

## Abstract

**Background:**

Posttraumatic stress disorder (PTSD) is known by a number of mental disorders, including recurring memories of trauma, mental appalling, and escaping of sign that make them recall the trauma in question. Clinical interviews serve as the main diagnostic tool for PTSD. With respect to treatment, either pharmacotherapy or psychotherapy or a combination of both is used as a therapeutic method for PTSD. In this study, a number of crucial genes related to PTSD, which can be considered as biomarker candidates, were represented.

**Materials and Methods:**

The genes related to PTSD were extracted from the STRING database and organized in a protein-protein interaction network with the help of Cytoscape software version 3.6.0. The network was analyzed, and the important genes were introduced based on central indices. The biological processes related to the crucial genes were enriched via gene ontology using ClueGO.

**Results:**

From a total of 100 genes, 63 genes were extracted that formed the main connected component, and of these, 12 crucial genes-*POMC, BDNF, FOS, NR3C1, CRH, IL6, NPS, HTR1A, NPY, CREB1, CRHR1,* and *TAC1*-were introduced. Biological processes were classified into the regulation of corticosterone, regulation of behavior, response to fungus, multicellular organism response to stress, and associative learning

**Conclusion:**

The introduced 12 crucial genes can be used as a biomarker panel related to PTSD and can be considered as a diagnostic reagent or drug target; however, more investigations are needed to use these genes as biomarkers.

## Introduction

Posttraumatic stress disorder (PTSD) is a condition that results from trauma. However, not all individuals exposed to a traumatic event develop PTSD [1]. A number of manifestations of PTSD, which include recurring remembrances of trauma, mental shocking, and escaping from the signs that make the people recall the trauma in question, have been reported [2]. Clinical interviews serve as the main diagnostic tool to determine PTSD [3].

Drug therapy or psychotherapy and a combination of both are used as therapeutic methods for PTSD [4]. Different molecular and cellular aspects of the disorder have been studied and discussed in detail [5-7]. The significant role of several hormones, such as cortisol, in PTSD is introduced and emphasized [8-10]. Genome-wide studies about PTSD have provided valuable information about the mechanism of the disease [11, 12].

In protein-protein interaction (PPI) network analysis, the genes, the production of genes and proteins or metabolites that are related to a condition are organized in an interactome unit based on graph theory [13, 14]. The elements (nodes) of the constructed network play various role in the network. The nodes that connect to a higher number of other elements of the network are known as hub nodes. These central nodes play a crucial role in the network. The absence of hub nodes leads to gross alteration in the topological properties of the network [15, 16]. Since there is a correlation between the network properties and the studied condition, the critical nodes of the network play a significant role in the pathology of diseases or disorders. The other important nodes in the network are known as the bottleneck nodes. These nodes control the other nodes of the network [17, 18]. Scientists had introduced more properties of the nodes such as closeness centrality and stress that identify the nodes as the more important nodes relative to other nodes [19]. Finally, network analysis can represent limited nodes among a large number of query nodes as the highlighted ones [20, 21]. Investigations show that these painted nodes are the main players related to the studied condition [22, 23]. It is possible to determine biological processes, molecular functions, and cellular components related to the critical genes (nodes) by enriching them through gene ontology (GO). The identified terms improve the understanding of the mechanism of condition or disease [24, 25].

A large number of diseases are studied via PPI network analysis, and the related crucial genes or proteins are identified and represented in a unique panel [26-30]. The findings can facilitate the introduction of efficient disease biomarkers. The discovered biomarkers are useful in treatment, diagnosis (especially in early diagnosis), and follow-up of patients [31-33]. The aim of this study was to determine the remarkable genes among the large numbers of PTSD-related genes via PPI network analysis.

## Materials and Methods

The genes related to PTSD were extracted from the STRING database. STRING

(http://string-db.org/) as an efficient interaction source is a plug-in of Cytoscape software. Cytoscape software and its applications such as STRING database are free sources that can be used to provide related proteins to diseases. This software is compatible with different sources. It is a useful tool for data collection and analysis using the PPI network.

The PPI network was constructed using Cytoscape software version 3.6.0. The main connected component of PTSD PPI network was analyzed by the network analyzer plug-in of Cytoscape. Since centrality parameters are the most important topological properties of the nodes of the PPI network, the 4 well-known central indices, including degree, betweenness, closeness, and stress of nodes, were considered to rank the nodes of the network. The numbers of top 20% of the genes base of degree values were selected as hub genes and 20% of genes based on betweenness were identified as bottleneck nodes. The third group of highlighted genes were the top 20% of high-score nodes based on closeness. Similar groups were chosen by stress values. The genes were classified into 5 categories on the basis of the following criteria and introduced as important nodes of the PPI network of PTSD:

1. The common genes between hub and bottleneck nodes (as hub-bottleneck genes)
2. The hub-bottleneck nodes that were painted in the high-score nodes based on closeness and stress
3. The common hub nodes with the selected genes via closeness and stress
4. The bottleneck genes with high scores of closeness and stress
5. The hub and bottleneck genes with high scores of closeness or stress

Connections between the important elements of the PPI network of PTSD were recognized by a subnetwork that was constructed by the critical nodes. The subnetwork was used as a screening tool to determine crucial genes. The regulatory pattern of the crucial genes was investigated via the literature survey for the validation of the findings. Finally, the crucial genes were enriched via GO by using ClueGO (http://apps.cytoscape.org/apps/cluego). The biological processes were classified and discussed in detail. The P-value of ≤0.01 was considered as the statistic index.

## Results

**Figure-1:**
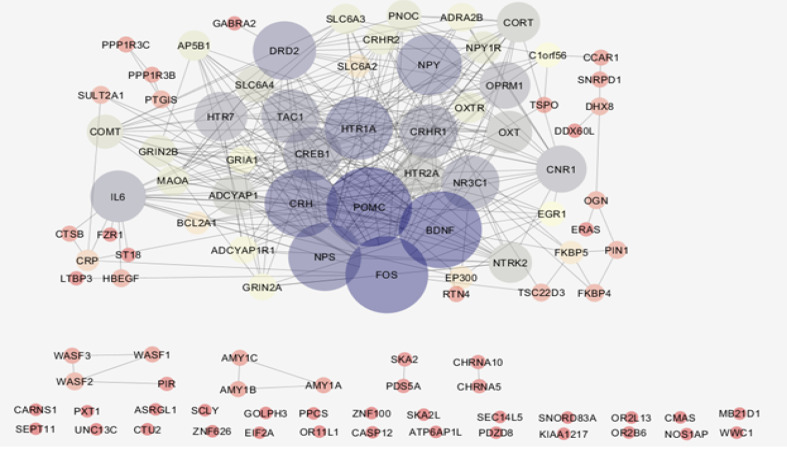
PPI network PTSD constructed by extracted 100 genes from STRING database is shown. The network includes one main connected component (contains 63 genes), a component containing four nodes, one component (inclosing three nodes), two paired components and 26 isolated nodes.

The genes related to a disease are required for constructing a PPI network. The data may be provided through an experimental study, literature survey, or database. In this study, the genes related to PTSD were extracted from the STRING database. As shown in Figure-1, a total of 63 genes were included in the main connected component. The network was analyzed, and the nodes were ranked on the basis of centrality parameters. Top 20% of nodes based on the degree value, betweenness centrality, closeness centrality, and stress were selected and organized in 4 groups (see Table-1). As described in the Materials and Methods section, 18 important genes were introduced; these are presented in Table-2.

**Table-1:** The Top 20% Nodes Related to the Main Component of PTDS PPI Network, Based On Degree Value, Betweenness Centrality, Closeness Centrality, and Stress Value

**Degree**	**Betweenness centrality**	**Closeness centrality**	**Stress**
POMC	BDNF	BDNF	BDNF
BDNF	IL6	FOS	IL6
FOS	FOS	POMC	FOS
CRH	FKBP5	CRH	POMC
NPS	ADCYAP1	NPS	NR3C1
HTR1A	POMC	CREB1	FKBP5
NPY	PIN1	TAC1	C1orf56
DRD2	C1orf56	NPY	PIN1
NR3C1	NR3C1	NR3C1	ADCYAP1
CREB1	OGN	CRHR1	CRH
CRHR1	PTGIS	IL6	OGN
TAC1	CCAR1	HTR1A	CCAR1

**Table-2:** The Most 18 Important Genes Related to PTDS

**Name**	**Description**	**Degree**	**Betweenness centrality**	**Closeness centrality**	**Stress**	**Disease score**
POMC	Proopiomelanocortin	32	0.10	0.56	1908	2.1
BDNF	Brain-derived neurotrophic factor	31	0.13	0.58	2120	2.6
FOS	FBJ murine osteosarcoma viral oncogene homolog	31	0.11	0.56	2060	2.0
NR3C1	Nuclear receptor subfamily 3, group C, member 1 (glucocorticoid receptor)	21	0.08	0.51	1806	2.5
CRH	Corticotropin releasing hormone	27	0.04	0.53	1124	2.7
IL6	Interleukin 6 (interferon, beta 2)	20	0.12	0.50	2068	1.5
NPS	Neuropeptide S	27	0.03	0.53	880	1.6
HTR1A	5-hydroxytryptamine (serotonin) receptor 1A, G protein-coupled	25	0.02	0.50	746	2.0
NPY	Neuropeptide Y	24	0.02	0.52	682	2.3
CREB1	Camp responsive element binding protein 1	21	0.05	0.52	976	1.4
CRHR1	Corticotropin releasing hormone receptor 1	21	0.04	0.51	934	2.5
TAC1	Tachykinin, precursor 1	21	0.03	0.52	890	1.2
FKBP5	FK506 binding protein 5	6	0.10	0.42	1748	3.0
ADCYAP1	Adenylate cyclase activating polypeptide 1 (pituitary)	15	0.10	0.48	1376	1.8
PIN1	Peptidylprolyl cis/trans isomerase, NIMA-interacting 1	3	0.09	0.32	1606	1.7
C1orf56	Chromosome 1 open reading frame 56	7	0.09	0.41	1638	1.8
OGN	Osteoglycin	3	0.07	0.25	1112	3.8
CCAR1	Cell division cycle and apoptosis regulator 1	2	0.06	0.31	1074	1.8

**Figure-2:**
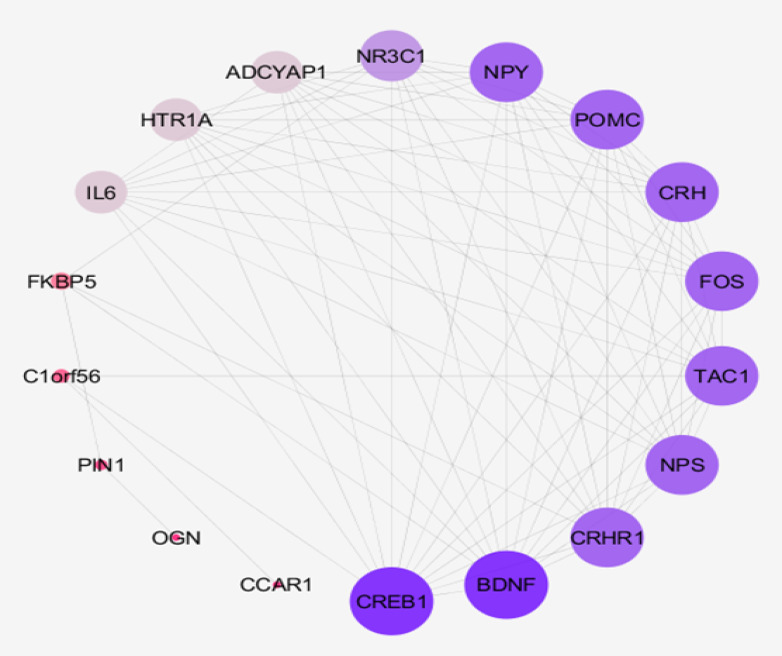
A sub-network including 18 top nodes of the main component of PTDS PPI network is presented

In Figure-2, connections between the important genes are highlighted via an integrative subnetwork. As shown in Figure-2, several important genes interact with almost all other nodes; however, a few genes have limited connections. As shown in Table-2, these few genes are bottleneck nodes that are common with the selected genes based on the stress value. Therefore, these nodes were excluded, and the remaining 12 nodes were introduced as the crucial genes related to PTSD (Table-3).

**Table-3:** Regulation Pattern for Elements of Displayed Sub-Network. Green and Red Colors Refer to Down and Up Regulation.

**Name**	**Down regulated**	**Up regulated**	**Reference**
**POMC**			Meyerhoff JL* et al* [34]
**BDNF**			Dell’Osso L *et al* [35]
**FOS**			Segman RH *et al* [36]
**NR3C1**			Vukojevic V *et al*[37]
**CRH**			Asalgoo S *et al* [38]
**IL6**			Gill J *et al* [39]
**NPS**			Ionescu IA *et al* [40]
**HTR1A**			Sullivan *et al* (41)
**NPY**			Cohen H *et al* [42]
**CREB1**			Segman RH *et al* [36]
**CRHR1**			Mehta D, Binder EB [43]
**TAC1**			Lindberg J [44]

**Figure-3:**
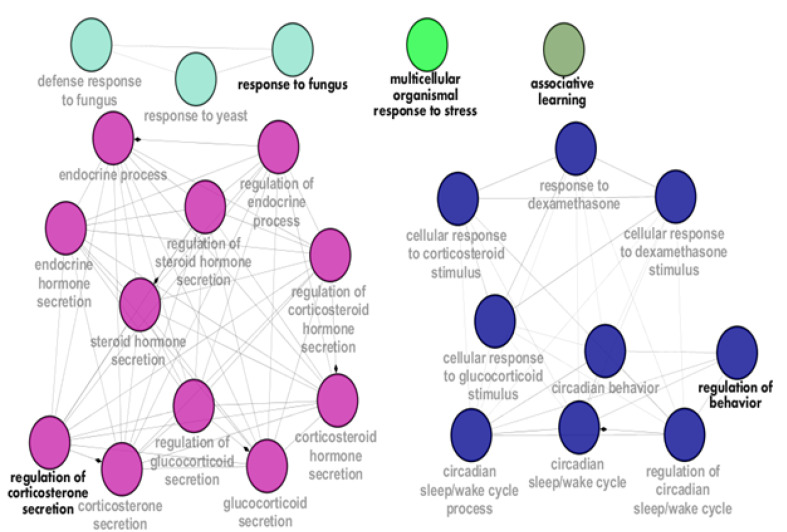
Gene ontology enrichment related to the 12 elements of table-3 via ClueGO is illustrated. The biological processes are categorized in the five groups including regulation of corticosterone, regulation of behavior, and response to fungus, multicellular organism response to stress, and associative learning. The terms were selected based on P≤ 0.01.

Expression change of the crucial genes in PTSD patients and animal models was investigated via the literature survey, and the findings are presented in Table-3. Because the attribution of a gene in biological processes is an important feature of the role of gene in the investigated disease, 12 crucial genes were enriched through GO, and the significant processes were determined as shown in Figure-3. Important roles of these biological processes in relation to PTSD are discussed in detail in the following section.

## Discussion

The PPI networks of diseases have different sizes, for example, esophageal adenocarcinoma has a small network compared with the pancreatic adenocarcinoma PPI network. The size of a network is in proportion to the number of the introduced genes. The more number of high-quality investigations provides more information and documents about a considered disorder. It seems that more investigations can explore more genes related to PTSD. Based on defined confidence, the genes are partly included in the network. In this study, by using the confidence value of 0.4 (default of software), 63% of the extracted nodes were included in the main connected component of the network. Analysis revealed that 18 genes play an important role in the PTSD PPI network. It is expected that these central genes be connected to each other and be organized in the integrated subnetwork. As shown in Figure-2, the numbers of nodes including FKBP5, PIN1, C1ORF56, OGN, and CCAR1 (the bottleneck nodes that are characterized with a high score of stress) have poor connections with the neighboring nodes. Corresponding with this finding, the mentioned group including 6 nodes is excluded, and the 12 remaining nodes were introduced as crucial nodes. In the following paragraphs, the role of these 12 crucial genes in the pathology of PTSD will be discussed briefly.

Proopiomelanocortin (*POMC*) is the first crucial gene related to PTSD. It is a hub-bottleneck node that is highlighted by high scores of closeness and stress. As is shown in Table-3, *POMC* gene is upregulated in PTSD. It can be processed to adrenocorticotropic hormone (ACTH) and melanocortin peptides [45].

Investigations indicate that an increase in ACTH concentration is accompanied by an increased value in cortisol concentration [46].

Cortisol, which is known as the “stress hormone,” is affected by inflammation, food intake, and obesity [47, 48]. It seems that *POMC* may be a suitable biomarker for PTSD.

The second crucial gene is *BDNF*, and its situation in the PPI network is similar to that of *POMC*. It is reported that brain-derived neurotrophic factor concentration decreases due to exposure to stress. This neurotrophic factor decrement is associated with physiological effects. Learning and memory are the two affected behavioral aspects related to *BDNF*. There are pieces of evidence that correspond to the role of *BDNF* in Alzheimer disease [49]. It can be concluded that the downregulation of *BDNF* in PTSD (see Table-3) is accompanied by the decrement of learning and memory. Cognitive aspects of PTSD were investigated. The finding indicates a decrease in sustained attention, working memory, and initial learning [50].

Similar to *POMC* and *BDNF*, the third gene, *FOS,* is a hub-bottleneck gene with high-scores of closeness and stress. *FOS*is known as a metabolite marker of tracing neuroanatomical connections and sites of action of neuroactive drugs [51].

This property of *FOS* is used to determine the involved brain nuclei in PTSD [52].

As shown in Table-3, *FOS* was upregulated in PTSD. It seems that *FOS* concentration is a suitable marker for the follow-up of patients.The fourth crucial gene is *NR3C1* that is upregulated, and its topological situation in the network is similar to the 3 aforementioned genes, which we have already discussed. Glucocorticoid hormones belong to a group of hormones that are secreted by the adrenal cortex and bind to glucocorticoid receptors in response to the stress via the circadian pattern. Hypothalamic corticotropin-releasing hormone (CRH) in response to the internal or external signals affects the secretion of pituitary hormone ACTH [53].

Cortisol is major human glucocorticoid [54]. The roles of ACTH and cortisol in PTSD were discussed in the initial part of the Discussion section. CRH is a hub node that is not a bottleneck gene, but it is highlighted by high values of closeness and stress (see Table-2). The closed relationship between CRH, ACTH, and cortisol refers to the critical role of each of them in PTSD. *CRHR1,* the familiarized gene in row 11 of Table-3, is another gene related to CRH, which encodes the R1 receptor of CRH. It is a hub node with a high closeness value (see Table-2). Its upregulation is consistent with the other related genes in Table-3. The relationship between a high level of interleukin (IL)-6, IL1β, interferon γ, and tumor necrosis factor (TNF α; inflammatory markers), and PTSD was reported by Passos *et al*. via a systematic review, meta-analysis, and meta-regression study [55].

As is represented in Table-3, IL6 is a unique bottleneck node that is achieved through high scores of closeness and stress. The roles of neuropeptide S (NPS) and neuropeptide Y (NPY) in alcohol use disorder are reported by Rodriguz and Covenas. These neuropeptides and corticotropin-releasing factors are responsible for the malfunction of brain in the patients [56]. The decrement of the NPY level in PTSD is reported, and it is suggested as a protector agent for PTSD [57].

Growing pieces of evidence indicate that NPY is a protective neurochemical reagent that is related to stress resilience [58]. These 2 hub nodes are tinted by high closeness values (Table-2). It is reported that there is a higher level of *HTR1A* in PTSD patients than in the healthy people. The patients were selected without comorbidity or major depressive disorder (MDD) [41].

The crucial roles of serotonin malfunction in MDD, anxiety disorders, and the overlap of these diseases with PTSD correspond to the important role of serotonin in PTSD [59]. The increased level of the serotonin receptor after the malfunction of serotonin can be interpreted in this regard. *HTR1A* is announced as a hub node with high closeness value (Table-2). cAMP-responsive element binding (CREB) protein 1 is a member of transcription factors that are involved in several neural processes such as stress response, learning, and neural plasticity. Investigation indicates that PTSD patients have less number of this protein relative to the healthy individuals [60]. As is reported, the significant role of CREB is neuronal caloric restriction [61]. Evidence indicates that TAC1 plays a main role in narcolepsy and the low level of this protein is recorded [44]. Association between PTSD and narcolepsy is reported and emphasized [62]. Biological processes related to the 12 crucial genes of the PPI network of PTSD correspond with the discussed roles of the nodes. As depicted in Figure-3, the regulation of corticosterone is an important process that is related to the nodes. A total of 11 terms are grouped in this cluster, including the regulation of the endocrine process, steroid hormone secretion, corticosteroid hormone secretion, and glucocorticoid secretion.

The second main cluster is the regulation of behavior that includes the number of terms that are correlated with the crucial nodes and have been discussed in detail. It seems that the analysis of the PPI network led to the finding critical opinions of PTSD. The introduced critical nodes can be used for the therapeutic method and diagnosis of PTSD and also for the follow-up of patients. The impact of the findings is to introduce a possible molecular-based method for the diagnosis of PTSD and also for the potential drug targets.

## Conclusion

In this study, at least 12 proteins among a large number of introduced proteins were identified, which can be used as a biomarker panel related to PTSD. The highlighted genes can be considered as the diagnostic reagents or drug targets; however, more investigation is needed to reduce this number to an economic quantity.

## Conflict of Interest

None declared
